# L‐PRF in extra‐oral wound care

**DOI:** 10.1111/prd.12605

**Published:** 2024-09-20

**Authors:** Nelson Pinto, Jize Yu, Sushil Koirala, Carlos Fernando Mourão, Catherine Andrade, Enrico Rescigno, Yelka Zamora, Diego Pinto, Marc Quirynen

**Affiliations:** ^1^ Center of Translational Medicine, Faculty of Medicine Universidad de la Frontera Temuco Chile; ^2^ Center for Research in Regenerative Medicine and Tissue Engineering Concepción Chile; ^3^ Faculty of Dentistry Universidad de Los Andes Santiago Chile; ^4^ Department of Oral Health Sciences KU Leuven & University Hospitals Leuven Leuven Belgium; ^5^ Department of Periodontology KU Leuven & University Hospitals Leuven Leuven Belgium; ^6^ Punyaarjan‐ Chronic Wound Healing Program Punyaarjan Foundation Kathmandu Nepal; ^7^ Department of Periodontology Tufts University School of Dental Medicine Boston Massachusetts USA; ^8^ Department of Periodontology and Implantology, Faculty of Dentistry Universidad de Los Andes Santiago Chile; ^9^ Department of Vascular Surgery Leonardi e Riboli Hospital Lavagna Genoa Italy; ^10^ Advanced Wound Care Clinic San José Costa Rica; ^11^ Resident in Orthopedic Surgery Hospital Traumatológico Concepción, Universidad de Concepción Concepción Chile

**Keywords:** chronic non‐healing ulcer, diabetic foot ulcer, extra‐oral wound, leprosy ulcer, leukocyte‐ and platelet‐rich fibrin, pressure ulcer, venous leg ulcer

## Abstract

Leukocyte‐ and platelet‐rich fibrin (L‐PRF), a by‐product of centrifuged autologous whole blood, contains high concentrations of platelets, leukocytes, and fibrin (the latter spontaneously creating a strong 3‐D network (a membrane)). L‐PRF membranes possess several characteristics essential in wound healing, including a barrier function, an antibacterial and analgesic activity, and the release of growth factors enhancing tissue regeneration and neo‐vasculogenesis. This review investigated the role of L‐PRF in treating non‐responding chronic wounds such as diabetic foot, venous leg ulcers, pressure ulcers, complex wounds, leprosy ulcers (Hansen's Disease), and other demanding wounds. Chronic wounds affect millions worldwide, negatively impacting their quality of life, productivity, and life expectancy while incurring high treatment costs for themselves and private and public health systems. L‐PRF has demonstrated clear adjunctive advantages in treating chronic skin wounds, shortening the time to complete wound closure, and improving patient‐reported outcome measures (including reducing pain and minimizing the need for analgesics). Also, in other demanding wounds, L‐PRF facilitates healing. To help clinicians, this article also proposes recommendations for the use of L‐PRF in the treatment of extra‐oral wounds.

## 1 INTRODUCTION

The human skin is a vital organ designed to develop multiple functions such as thermoregulation, vitamin D metabolization, detection of sensory stimuli, as well as responding to mechanical trauma, chemical reagents, and pathogens. It is necessary to preserve its structural integrity to maintain its functionality. Ruptures of the skin layers or adjacent tissues bring anatomical and functional changes, resulting in increased morbidity.^1^

Wound healing after an injury is a complex process that follows a consistent pattern across various tissues. It involves numerous cellular and humoral components, such as the local influx and activation of macrophages and platelets. Platelets are cytoplasmic fragments of megakaryocytes formed in the bone marrow. They contain, in their α granules, protein growth factors with a capital role in hemostasis and wound healing, including: CTGF (conjunctive tissue growth factor), EGF (epidermal growth factor), FGF (fibroblast growth factor), IGF‐1 (insulin growth factor), PDGF (platelet‐derived growth factor), TGF (transforming growth factor), and VEGF (vascular endothelial growth factor). After activation, exogenous or endogenous, platelets secrete these growth factors which will bind to transmembrane receptors on target cells (e.g., undifferentiated mesenchymal cells, osteoblasts, fibroblasts, endothelial cells and epidermal cells), triggering effects such as cell proliferation, angiogenesis, synthesis of collagen and extracellular matrix components, and reducing apoptosis, as such initiating wound healing and tissue repair.^2^, ^3^

Leucocyte‐ and platelet‐rich fibrin (L‐PRF), a 2nd generation platelet concentrate, with, compared with whole blood, a 20‐fold higher concentration of platelets and leucocytes entrapped in a strong 3‐D fibrin network,^4^ releases all factors mentioned above in a sustained manner for 7–14 days.^5^, ^6^, ^7^, ^8^, ^9^, ^10^ L‐PRF also has antibacterial properties exhibiting strong activity, comparable with gentamicin and oxacillin, against methicillin‐susceptible *Staphylococcus aureus* (MSSA), methicillin‐resistant *Staphylococcus aureus* (MRSA),^11^, ^12^, ^13^, ^14^, ^15^ and even *Escherichia coli*.^16^, ^17^, ^18^ The plethora of capacities of L‐PRF also includes a fibrous structure conducive to cell attachment and migration, enhanced angiogenesis, analgesic activity, the promotion of stem cell differentiation towards osteoblasts, and anti‐inflammatory activity. Moreover, L‐PRF, thanks to its physical characteristics, can act as an optimal biological wound dressing, protecting the wound from proteolysis. The biological background of L‐PRF has been discussed more in detail in several recent reviews,^3^, ^19^, ^20^ which are part of this special issue on autologous platelet concentrates (APC).

Acute wounds heal through an orderly and timely reparative process (with overlapping phases of inflammation, epithelialization, fibroplasia, and maturation), that restores the anatomic and functional integrity.^3^, ^21^, ^22^ Chronic wounds, on the other hand, fail to progress within a specific timeframe despite appropriate care, due to an inability to proceed through the normal stages of healing. Moreover, they cannot be repaired in an orderly way, so a sustained anatomic and functional result is not achieved.^23^ The normal physiology is transformed into the pathophysiology of a chronic cycle without a distinct wound closure endpoint. Chronic wounds are often associated with underlying medical conditions such as diabetes, venous or arterial insufficiency, or local disorders (e.g. persistent localized pressure) and frequently affect the lower extremities.^24^

Diabetes mellitus is an important chronic illness that affects a growing population (currently 422 million people in the world, US Department of Health and Human Services 2020).^25^ It considerably raises the likelihood of multiple complications, including the development of a diabetic foot ulcer (DFU), a common, highly morbid, and costly condition.^26^ Lipsky et al.^27^ estimated that 25% of the diabetic population will develop a DFU during their life. More than 50% of DFUs become infected, of which 20% result in amputations.^28^ Moreover, patients with DFU experience a substantial decline in quality of life and are more likely to incur significant financial losses.^29^, ^30^

Venous leg ulcers represent between 60% and 80% of all leg ulcerations.^31^, ^32^ Their three months healing rate is estimated at 40%, and once healed, up to 80% of patients develop a recurrence within three months.^32^, ^33^, ^34^ The prevalence of VLUs is reported to be ~1.1%.^32^, ^35^, ^36^

Pressure ulcers (PU), also known as decubitus ulcers, are ischemic skin lesions that disrupt the integrity of the skin and underlying tissues with substance loss. A meta‐analysis by Li et al.^37^ estimated the global prevalence of pressure ulcers in hospitalized adult patients (≥ 16 years), to be 12.8%, with a hospital‐acquired pressure injury (HAPI) incidence of 8.4%. In the United States, a relative though not statistically significant decrease in the incidence of pressure injuries was observed between 1990 and 2017.^38^

Leprosy (LU), or Hansen's disease, is caused by *Mycobacterium leprae* or *Mycobacterium lepromatosis*.^39^ The first manifestation of leprosy is often the appearance of hypopigmented, anesthetic skin patches. If not diagnosed and treated promptly, the disease can be progressive and permanently damage the peripheral nervous system, the soft tissue of the nose and throat, and vision. Due to a loss of sensation in the extremities, people easily injure themselves. These often painless injuries can become infected, which may eventually result in tissue loss in affected areas such as the fingers or toes.^39^ Leprosy mainly affects people in resource‐poor countries and is closely linked to health inequalities and poverty.^40^ In 2019, the number of new leprosy patients reported globally was 202.185.^41^ Plantar ulcers, which occur in about 10%–20% of patients, are one of the commonest complications of leprosy leading to Grade 2 disability.^42^

This systematic review aimed to analyze the literature to verify the potential benefits of L‐PRF (including all its modifications: A‐PRF, A‐PRF+, T‐PRF, H‐PRF, CGF) in the treatment of chronic wounds, especially DFU, VLU, PU, as well as LU.^43^ The adjunctive role of L‐PRF in the healing of other demanding wounds was also explored. Aside from aspects such as wound closure, patient‐reported outcome measures (PROMs) such as healing time, wound recurrence, pain relief, need for analgesics, etc. were also considered. Finally, based on the authors' experience, recommendations are proposed for applying L‐PRF in chronic wounds.

## 2 LITERATURE SEARCH

For this review, the following search query was entered in PubMed: chronic wounds OR ulcer OR cutaneous ulcer OR lower limb OR VLU OR DFU OR venous leg ulcer OR diabetic ulcer OR diabetic foot OR pressure ulcer OR non‐healing ulcer OR leprosy OR Hansen's disease AND PRF OR L‐PRF OR platelet‐rich fibrin OR A‐PRF OR CGF OR concentrated growth factor OR H‐PRF OR T‐PRF. After initial screening of titles and abstracts by two independent reviewers (JY and MQ), 21 studies, from the 99 hits, were identified as useful. To ensure literature saturation, we scanned the reference lists of all studies and additional review articles, leading to another 10 articles. After removing case reports with less than 6 patients and articles on “non‐chronic wounds,” 23 studies remained, all reporting on the impact of L‐PRF (including all its modifications such as A‐PRF, A‐PRF+, T‐PRF, H‐PRF, CGF). From these 23 included studies, 9 were designed as randomized clinical trials (RCT), 3 as case control (CCS), 9 as prospective, and 2 as retrospective studies. Most articles (11) reported on a mixture of chronic wounds, the others on a specific wound type (3 on DFU, 1 on DFU with gangrene, 4 on VLU, 4 on LU).

### 2.1 L‐PRF in the healing of chronic wounds (Table 1)

#### 2.1.1 Studies including various types of chronic ulcers

Eleven articles reported on various chronic wounds,^44^, ^45^, ^46^, ^47^, ^48^, ^49^, ^50^, ^51^, ^52^, ^53^, ^54^ representing 3 RCT,^44^, ^46^, ^52^ 1 CCS,^54^ 5 prospective case series (Ps),^45^, ^47^, ^50^, ^51^, ^53^ and 2 retrospective case series (Rs).^48^, ^49^ Only 3 of these articles presented sub‐data per wound type.^47^, ^49^, ^50^

**TABLE 1:** Most relevant clinical trials on the impact of L‐PRF on chronic wounds.

Author	Study: type follow‐ up	Subjects: gender age range % smokers	Wound type wound age	Centrifuge RPM g force minutes	Intervention P^r^ = prior to treatment R/ = treatment	Wound resolution (CR = complete resolution) Wound recurrence PROMs
**Chronic ulcers of varies etiologies**						
Pravin et al. 2016^44^	RCT 12 w ♦	♀ = 10/♂ = 20 $\bar{x}$ age: T^1^: 44 y T^2^: 47 y Smoker: NR	30 non‐healing ulcers of various aetiologies: 2 DFU, 22 VLU, 1 TU, 4 TrU, 1 vasculitis *2–12 m*	PRP: Double spin L‐PRF	P^r^: NR R/maximum 6 applications: 15 T^1^: weekly PRP^inj^ 15 T^2^: weekly L‐PRF^cl^	L‐PRF gave: **Faster healing** ($\bar{x}$ duration T^1^ = 6.5 vs. T^2^ = 5.7 w) **>CR** (8/15 in T^1^, 11/15 in T^2^ after 6 w) No recurrence of wounds No AE except some rare and mild pain
Rescigno et al. 2016^45^	Ps 8 w ♦	♀ = 12/♂ = 5 Age: 23–99 y Smoker: NR	22 non‐healing ulcers of various aetiologies: 6 DFU, 2 VLU, 10 TU, 1 PU, 3 others	IntraSpin 2700 rpm/12′ ~400 g	P^r^: adv dress R/thorough cleaning & debridement, L‐PRF^cl^ weekly	CR in 19/22 lesions 2 p discont'd AE: in 1 patient the lesion worsened
Shreyas et al. 2017^46^	RCT 8 w ♦	♀ = 17/♂ = 33 $\bar{x}$ age: T: 35 ± 16 y C: 41 ± 17 y 2% smoker	50 non‐healing ulcers: 1 VLU, 1 PU, 29 TU, 19 infection $\bar{x}$ *size 12 cm* ^*2*^ *11–12 w*	Remi R‐8C 3000 rpm/10′ ~ 704 g	P^r^: NR R/ up to 6 w: thorough cleaning & debridement, 25 T: L‐PRF^cl*^ + n‐ad dress weekly 25 C: paraffin mesh + n‐ad dress (changed every 48 h)	L‐PRF gave: Less presence of exudate from w 2 on **Faster healing from d 2** After 6 w (T: 97% vs. C: 82% wound size reduction) **Faster CR (T**: **3.5 w vs. C**: **4.2 w)**
Pinto et al. 2018^47^	Ps 1 y	♀ = 18/♂ = 26 $\bar{x}$ age: 64 ± 14 y 7% smokers	49 non‐healing ulcers: 10 DFU, 32 VLU, 5 PU, 2 Compl	IntraSpin 2700 rpm/12′ ~ 400 g	P^r^: optimal standard care R/thorough cleaning & debridement, L‐PRF^m^ weekly until complete wound closure	17 VLU size ≤10 cm^2^: 100% CR; 15 VLU size > 10 cm^2^: 100% CR in 10; 5 discont'd before CR 10 DFU: $\bar{x}$ size 6.7 ± 8.2 cm^2^: 100% CR 5 PU: $\bar{x}$ size 5.4 ± 4.8 cm^2^: 100% CR in 2, 3 discont'd before CR Compl 2.4 and 4.7 cm^2^: 100% CR Healing time proportional to original wound size For VLU: pain reduction from w 3, ∅ after m 3 No bad smell No recurrence of wounds during 1st year after therapy No AE
Ozer & Colak 2019^48^	Rs 30 m ♦	♀ = 6/♂ = 11 Age: 18–77 y Smoker: NR	17 non‐healing compl wounds of lower extremities: *Size: 2–63 cm* ^*2*^ *2 m–12* y	— — ~1630 g/5'	P^r^: # conditions R/ L‐PRF^cl*^ twice/w until complete wound closure	100% CR after a $\bar{x}$ time of 12 w, ranging 4–30 w Healing time proportional to original wound size No correlation between wound duration and healing time No wound recurrence for at least 6 m after therapy No AE
Bilgen et al. 2021^49^	Rs 6 m	♀ = 7/♂ = 9 Age: 36–82 y Smoker: NR	16 non‐healing ulcers: 8 DFU, 3 VLU, 2 TU, 3 PU *1 m‐15 y*	Medwelt 3000 rpm/10' ~805 g max	P^r^: conventional treatment R/ L‐PRF^m^ ± weekly until complete wound closure	$\bar{x}$ number of L‐PRF applications: 4.4 (range 1–8) DFU: 3.8, TU1.5, VLU: 4.7, PU: 7.3 $\bar{x}$ recovery time 2.1 m (10 d–4 m), 63% CR within 4 applications No wound recurrence during 1st 6 months after therapy No AE
Dorjay & Sinha 2021^50^	Ps 3 m	♀ = 9/♂ = 9 Age: 29–55 y Smoker: NR	18 non‐healing ulcers: $\bar{x}$ *size 8.4 cm* ^*2*^ 2 DFU, 3 VLU, 4 Neu, 2 TU, 6 LU 1 post skin graft *Range: 2.5–12 m*	— 2000 rpm/10′	P^r^: conventional treatment R/ thorough cleaning, L‐PRF^m^ weekly until complete wound closure	$\bar{x}$ number of L‐PRF applications: DFU (*19 cm* ^*2*^): 6.0, VLU (*10 cm* ^*2*^): 4.3, LU (*5.3 cm* ^*2*^): 3.2, Neuropathic (*6.8 cm* ^*2*^): 4 100% CR in 16 non‐diabetic patients in 3–7 w 100% CR in 2 diabetic patients in 7–9 w 1 partial recurrence after 6 m Healing time proportional to original wound size No AE
Helmy et al. 2021^51^	Ps 8 w ♦	♀ = 8/♂ = 42 Age: 20–65 y 40% smokers	50 non‐healing ulcers: 24 DFU, 22 VLU, 4 TU No active infections *2–6 m*	— 3000 rpm/10′ —	P^r^: optimal standard care R/ up to 8 w: Surgical & chemical debridement, L‐PRF^m^ weekly	18/50 CR within 8 w Wound size reduction after 8 w (from 7.2 to 1.0 cm^2^)
Singampalli et al. 2022^52^	RCT 6 w ♦	♀ = 20/♂ = 30 $\bar{x}$ age: 43 ± 3 y 0% smokers	50 non‐healing ulcer of lower limb: 12 DFU, 22 VLU, 9 TU, 7 TrU *>12 w*	— 3000 rpm/15′ —	P^r^: surgical debridement & thorough saline washing R/ for 6 w: 25 T: L‐PRF^cl*^ dress weekly 25 C: saline dress weekly	L‐PRF gave: **Faster healing** (wound size reduction after 6 weeks) T: from 15.0 to 1.6 cm^2^ vs. C: from 13.3 to 11.1 cm^2^ >CR (T: 24/25 in 6.6 w; C: NR) Healing time proportional to original wound size No AE
Madhu et al. 2022^53^	Ps 4 w ♦	♀ = 8/♂ = 42 $\bar{x}$ age: 44 ± 14 y Smoker: NR	50 non‐healing ulcers: 12 DFU, 8 PU, 6 TU, 16 Le, 8 TrU *> 6 w*	— 3000 rpm/10′ —	P^r^: NR R/ for 4 w: thorough debridement and cleaning, L‐PRF^m^ dress weekly	Wound improvement (at 3 w): 6 good, 20 moderate, 24 mild *6/50: 50%–70% improvement* *20/50: 25%–50% improvement* *24/50: <25% improvement*
Onwuagha et al. 2023^54^	CCS 2 m ♦	♀ = 13/♂ = 41 Age: 18–65 y 19% smokers	Primarily non‐healing ulcers: 16 DFU, 2 DHU, 6 VLU, 8 TU, 5 Scu, 7 burns, 7 post inf, 3 post‐sur *1 y*	Eschmed 800D 3000 rpm/10′ —	P^r^: NR R/ up to 8 w: saline cleaning 27 T: sofratulle + L‐PRF^cl*^ dress weekly 27 C: sofratule +2% povidone iodine solution dressing weekly	L‐PRF gave: **Less infection/slough** from w2 on **Less wound exudate** from w2 on **Faster re‐epithelialization** from w2 on **>healthy granulation tissue** from w2 on **>wound size reduction** T: from 4.5 to 3.1 cm^2^ versus C: 4.6 to 4.2 cm^2^ **> reduction in foul odor** from w2 on **< cost of dressing** **< pain** after 2w no AE
**Diabetic foot ulcer**						
Wang et al. 2024^56^	CCS 5 w	42 patients NR Smoker: NR	DFU $\bar{x}$ *size*: T: 8.6, C: 8.2 cm^2^ *> 1 w*	USTC 3000 rpm/20′ —	P^r^: surgical debridement & infection prevention R/ for 5 w: 21 T: L‐PRF^cl*^ weekly 21 C: mupirocin ointment + rhEGF gel twice/w	L‐PRF gave: **>wound size reduction**: at w 1, 2, 3, 4, 5 T: 12%, 59%, 84%, 88%, 93% vs. C: 7%, 37%, 56%, 78%, 75% **>CR**: at w 5: T: 20/21, C: 12/21
Kartika et al. 2021^55^	RCT 7 d	♀ = 18/♂ = 12 $\bar{x}$ age T1: 60 ± 13 y T2: 65 ± 12 y C: 66 ± 12 y Smoker: NR	Non‐healing DFU $\bar{x}$ *size*: <40 cm^2^ *3 m*	— — ~200 g/8′	P^r^: NR R/ 1x application: 10 T^1^: A‐PRF^m^ + HA 10 T^2^: A‐PRF^m^ 10 C: 0.9% NaCl + for all a protective bandage	A‐PRF with or without HA gave: **> granulation index** at d3 (T^1^: 83 vs. T^2^: 68 vs. C: 60) **> granulation index** at d7 (T^1^: 97 vs. T^2^: 82 vs. C: 66) **>VEGF** at d3 (T^1^: 43 vs. T^2^: 2 vs. C: 5 pg/mg) **>VEGF** at d7 (T^1^: 276 vs. T^2^: 105 vs. C: 28 pg/mg) **<IL‐6 levels** at d3 (T^1^: 11 vs. T^2^: 4 vs. C: 4 pg/mg) **<IL‐6 levels** at d7 (T^1^: 18 vs. T^2^: 8 vs. C: 36 pg/mg) *With superior effects of A‐PRF with HA*
Wang et al. 2023^57^	CCS 1 y	♀ = 21/♂ = 29 $\bar{x}$ age: 53 ± 4 y Smoker: NR	DFU $\bar{x}$ *size*: 10 cm^2^ *>1 w*	Anhui USTC — ~1006 g/20′	P^r^: thorough debridement R/prior to skin grafting 25 T: L‐PRF^cl^ every 2 days 25 C: Vaseline^gauze^ every 2 days *Both until reaching standard for skin grafting*	L‐PRF gave: **Shorter time to skin grafting** (T: 6 d vs. C: 12 d) **Less skin graft necrosis** (T: 7/25 vs. C: 16/25) **Less postoperative infection** (T: 6/25 vs. C: 14/25) **Less ulcer recurrence** (1 y follow‐up; T: 4/25 vs. C: 9/25) **Lower need for amputation** (T: 5/25 vs. C: 12/25) **Better foot scores** (T: 95 vs. C: 89)
**Diabetic foot with gangrene/osteomyelitis**						
Crisci et al. 2023^58^	Ps Average 19 m	♀ = 2/♂ = 5 $\bar{x}$ age: 65 ± 5 y Smoker: NR	Ulcerative osteomyelitis of diabetic foot *Average 10 w*	Duo Quattro 1300 rpm/8′ ~ 189 g	P^r^: surgical debridement, removal of bone particles R/ 1–2 applications: Cleaning with H_2_O_2_ and iodopovidone, A‐PRF^mF^ + A‐PRF^ex^ in wound In combination with antibiotics	100% CR within 31.4 ± 4.5 d in 6/7 patients No wound recurrence (1 up to 6 y)
**Venous leg ulcer**						
Somani & Rai 2017^59^	RCT 4 w	*n* = 15 Smoker: NR	Non‐healing VLU *Size*: 1–5 cm^2^ *>6 m*	— 3000 rpm/15' —	P^r^: NR R/ for 4 w: 9 T: L‐PRF^cl*^ weekly 6 C: saline dressing weekly	L‐PRF gave: **>reduction in ulcer size**: T: 26%, 46%, 77%, 86% vs. C: 15%, 23%, 35%, 43% after 1, 2, 3 4 w **>CR**: T: 5/9 vs. C: 0/6 after 4 w No AE
Goda 2018^60^	RCT 8 w	♀ = 17/♂ = 19 $\bar{x}$ age: 40 ± 7 y 50% smokers	VLU *NR*	— 3000 rpm/10′ —	P^r^: NR R/ up to 8 w: 18 T: L‐PRF^m^ + paraffin^gauze^ weekly 18 C: paraffin^gauze^ every 2 d	L‐PRF gave: **>Reduction in ulcer size for VLU ≤ 10 cm** ^**2**^: T: 25%, 53%, 80%, 100% versus C: 12%, 24%, 47%, 62% after 1, 2, 3, 4 w **>Reduction in ulcer size for VLU > 10 cm** ^**2**^: T: 16%, 31%, 49%, 64%, 80%, 96%, 100% vs. C: 7%, 14%, 29%, 43%, 58%, 72%, 87% after 1, 2, 3, 4, 5, 6, 7 w **Faster CR for VLU ≤ 10 cm** ^**2**^ (T:4 w vs. C: 6 w) **Faster CR for VLU > 10 cm** ^**2**^ (T: 7 w vs. C: >8 w) No AE
Amato et al. 2019^61^	RCT 24 w	♀ = 64/♂ = 36 $\bar{x}$ age: 68 ± 8 y Smoker: NR	VLU $\bar{x}$ *size*: 24 cm^2^ *NR*	Medifuge # settings/14′ + heparin	P^r^: NR R/ for up to 12 w: 53 T: CGF^gel^ + HA^gauze^ weekly 47 C: HA^gauze^ weekly	L‐PRF gave: **Faster CR** T: 11 at 5, 14 at 6, 10 at 7, 8 at 8, 7 at 9, 2 at 10, 1 at 11 w C: 2 at 5, 1 at 6, 3 at 8, 5 at 9, 7 at 10, 6 at 11, and 8 at 12 w **<pain** from w 2 on No AE
Yuvasri & Rai 2020^62^	RCT 4 w	20 patients NR Smoker: NR	VLU *>6 m*	— 3000 rpm/15′ —	P^r^: NR R/ for 4 w: 10 T: L‐PRF^cl*^ weekly 10 C: Unna's paste weekly	L‐PRF gave: Similar wound size reduction: T: 8%, 35%, 62%, 86% versus C: 15%, 30%, 56%, 72% after 1, 2, 3, 4 w >CR: T: 4/10 versus C: 0/10
**Leprosy ulcer**						
Nagaraju et al. 2017^42^	Ps NR	♀ = 2/♂ = 5 $\bar{x}$ age: 38 y Smoker: NR	9 non‐healing Leprosy ulcer NR *2–12 m*	— 3000 rpm/10′ ~ 400 g	P^r^: optimal standard care R/≤5 applications: L‐PRF^m^ weekly	93.5% reduction in wound size after 2 applications $\bar{x}$ number of applications for CR = 3 No AE
Ghatge et al. 2021^64^	Ps 6 w	♀ = 7/♂ = 18 $\bar{x}$ age: 51 y Smoker: NR	Trophic ulcer secondary to Leprosy —	— 3000 rpm/10′ —	P^r^: conventional treatment R/ up to 6 w: Cleaning with betadine and saline, L‐PRF^cl*^ weekly	CR in 17/25 after 6 w Wound volume reduction after 6 w from 2.5 to 0.3 cm^3^ No correlation between wound duration and healing time No AE
Dorjay et al. 2022^65^	Ps 1 y	♀ = 3/♂ = 7 Age: 35‐60 y Smoker: NR	Non‐healing leprosy ulcers 2–12 cm^2^, $\bar{x}$ *size*: 6.2 cm^2^ *3–12 m*	— 2000 rpm/10′ —	P^r^: optimal standard care R/ Thoroughly cleaning with povidone iodine and saline, L‐PRF^m^ weekly	CR after 4–6 applications No AE No recurrence after 1 y
Ratan et al. 2023^63^	RCT 8 w	♀ = 27/♂ = 15 Age: 21‐60y Smoker: NR	Primarily leprosy ulcers 0.5–10 cm^2^ >6 w	PRP: Double spin L‐PRF: 3000 rpm/10′	P^r^: — R/ up to 6 w (weekly): Cleaning with antiseptic 21 T^1^ PRP injection (wound margin) 21 T^2^ L‐PRF^cl^ + both n‐ad dress	CR T^1^ = 19; T^2^ = 20 Number of dressings up to re‐epithelization: T^1^ 3.2; T^2^ 3.5 Infection: T^1^ = 2; T^2^ = 1 Ulcer recurrence: T^1^ = 3; T^2^ = 4

*Note*: Data in bold indicate a statistically significant difference between test and control in case of an RCT.

Abbreviations: DFU, diabetic foot ulcer; PU, pressure wound, leprosy wound, and other types of chronic lesions; VLU, venous leg ulcer.

**Study type**: ♦, study without sub analyses per wound type; CCS, case control study; d, day; m, month; Ps/Rs, prospective/retrospective case series; RCT, randomized clinical trial; w, week; y, year. **Subjects**: ♀, female; ♂, male; $\bar{x}$ age, mean age. **Wound info**: Compl, complex wound; DFU, diabetic foot ulcer; DHU, diabetic hand ulcer; inf, infection; LU, leprosy ulcer/Hansen's disease; Neu, neuropathic; PU, pressure ulcer; ScU, sickle cell ulcer; sur, post‐surgery wound breakdown; TrU, trophic wound; TU, trauma ulcer; VLU, venous leg ulcer. **Centrifugation data**: device—g, g‐force; min., minutes; rpm, revolutions/rotations per minute. **Intervention**: *, often compressed in situ; /^cl^, clot; /^ex^, exudate; adv dress, advanced dressing; C, control; HA, hyaluronic acid; L‐PRF^m^, L‐PRF membrane; ^mF^, face part of membrane; n‐ad dress, non‐adherent dressing; P^r^, prior to start L‐PRF treatment; R/, treatment: (debridement often only once at start therapy); rhEGF, recombinant human epidermal growth factor gel; T, test. **Outcome**: Data in bold for RCTs and CCS, statistically different; AE, adverse events; wound closure—<, decreased; >, increased; CR, complete resolution; discont'd, discontinued; PROMs, patient‐reported outcome measures; Ø, none; x– = mean.

##### Randomized clinical trials

Pravin et al.^44^ compared (in 2× 15 patients, various non‐healing ulcers) the outcome of PRP (injection) with L‐PRF (clot) and observed significantly faster healing for the latter. Shreyas et al. compared (in 2× 25 patients, various non‐healing chronic wounds) the application of a paraffin mesh (changed every 2nd day) with an L‐PRF clot (weekly renewed), both covered with a non‐adherent dress. The L‐PRF clot resulted in significantly faster healing (97% vs. 82% wound size reduction after 6 weeks), and an earlier complete wound resolution (CR) at 3.5 versus 4.2 weeks.^46^ Singampalli et al.^52^ compared (in 2× 25 patients, different non‐healing wounds) the impact of an L‐PRF clot wound dressing with the application of a saline dressing, and observed a statistically significant faster wound healing with L‐PRF (after 6 weeks: a wound reduction from 15.0 to 1.6 cm^2^ for L‐PRF vs. from 13.3 to 11.0 cm^2^ for the saline dressing).

##### Overall observations considering all 11 studies

A significant heterogeneity was obvious between the 11 studies. A large variety of wounds were included (primarily DFU, VLU, PU, LU, but also some trauma ulcers (TU), neuropathic ulcers, trophic wounds (TrU), vasculitis ulcers, as well as complex wounds). L‐PRF membranes (5 studies) as well as clots (6 studies) have been used, but the latter clots are, of course, compressed within the wound when the final dressing is applied. In 10/11 studies, the L‐PRF matrices were reapplied weekly, except in the study by Ozer & Colak^48^ where they were replaced every 2nd day. The number of L‐PRF applications varied from 4 weeks until complete resolution (CR) was reached. In most trials, both the medical condition of the patient and potential causative factors were treated prior to or during the regenerative treatment. In nearly all studies, patients were included in whom a previous therapy (often called standard or conventional therapy) had failed for a longer period of time, ranging from 4 weeks to 12 years. In other words, the patients served as their own control group (auto‐controlled studies).

Wound size reduction was observed in 287/288 wounds; only in one patient was the treatment interrupted because the wound worsened.^45^ Complete wound resolution (CR) was obtained in 81.7% of the cases in studies of 6 weeks or longer. In studies that ran until CR, the treatment time/number of weekly applications varied (5.7 weeks: Pravin et al. 2016; 3.5 weeks: Shreyas et al. 2017; 12 weeks: Ozer & Colak 2019; 4.4 applications: Bilgen et al. 2021; 3–9 weeks: Dorjay & Sinha 2021; and 6.6 weeks Singampalli et al. 2022; as overall means).^44^, ^46^, ^48^, ^49^, ^50^, ^52^ The healing time was clearly proportional to the original wound size^47^, ^48^, ^50^, ^52^ and Ozer & Colak 2019^48^ reported that there was no correlation between the duration of the wound before L‐PRF treatment and the time to CR after L‐PRF therapy.

**FIGURE 1:**
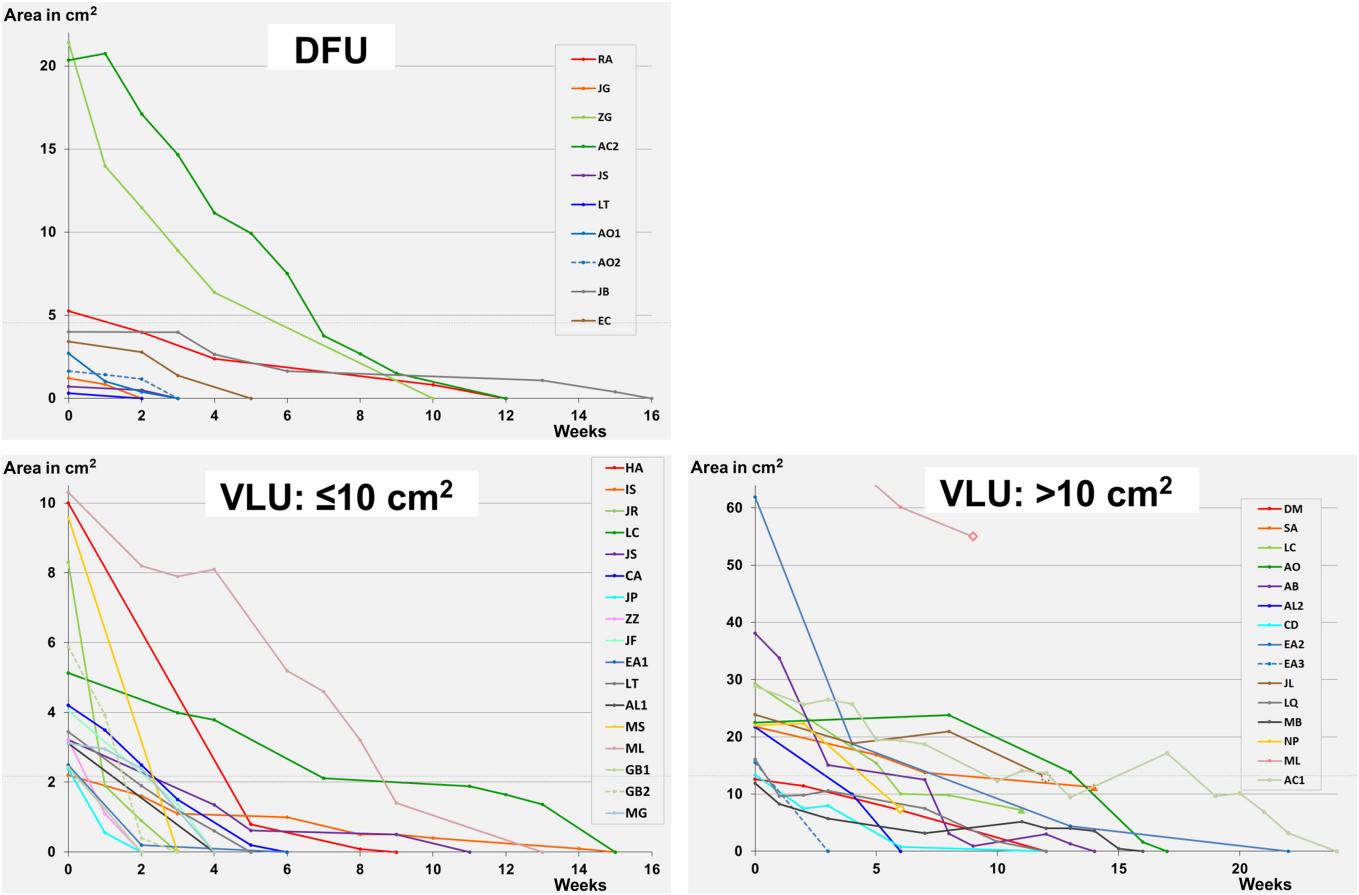
Reduction in wound area (expressed in cm^2^) over time for: DFUs (9 patients, 10 wounds) and VLUs (subdivided for ≤10 cm^2^ (16 patients, 17 wounds) and larger >10 cm^2^ (14 patients, 15 wounds) initial wounds (patient ML started with an initial wound area of 74.5 cm^2^, but to have a better view on the other wounds, the *y*‐axis was cut off at 64 cm^2^). L‐PRF was applied weekly, and pictures to analyze changes in wound area were taken at some weeks' interval (each mark represents a wound size analysis). For patients who did not reach full wound closure, the reason was mentioned (Δ: Interruption because of financial reasons, Ο: Moved to different area/hospital, or ◊: No info).

Figure 1 illustrates more in detail the healing of initial non‐responding DFU as well as VLU once L‐PRF membranes were applied.^47^ Thirty‐seven consecutive patients with VLUs (*n* = 28, 32 wounds: 17 ≤ 10 cm^2^ and 15 > 10 cm^2^), DFUs (*n* = 9, 10 wounds), all refractory to a standard treatment for ≥3 months, received a weekly application of L‐PRF membranes. All VLUs ≤10 cm^2^ and all DFUs showed full wound closure within 3 months. All VLUs >10 cm^2^ who continued therapy (10 wounds) could be closed, whereas a clear improvement in wound size was observed in the five patients who discontinued their therapy.

Five studies explored the recurrence of the ulcers the first 6–12 months after L‐PRF therapy, with all together 1 partial recurrence on 107 treated non‐healing chronic wounds,^44^, ^47^, ^48^, ^49^, ^50^ of course taking into consideration that also the cause of the ulcer was treated by improving the medical condition of the patient. Other reported beneficial PROMs included: a reduction in wound exudate from the 2nd week on,^46^, ^54^ a reduction in foul odor, also from the week 2nd on,^47^, ^54^ as well as less pain, lower need of analgesics, and less infection from weeks 2–3 on.^47^, ^54^

#### 2.1.2 Studies reporting on “one specific” chronic ulcer

##### Diabetic foot ulcer

**FIGURE 2:**
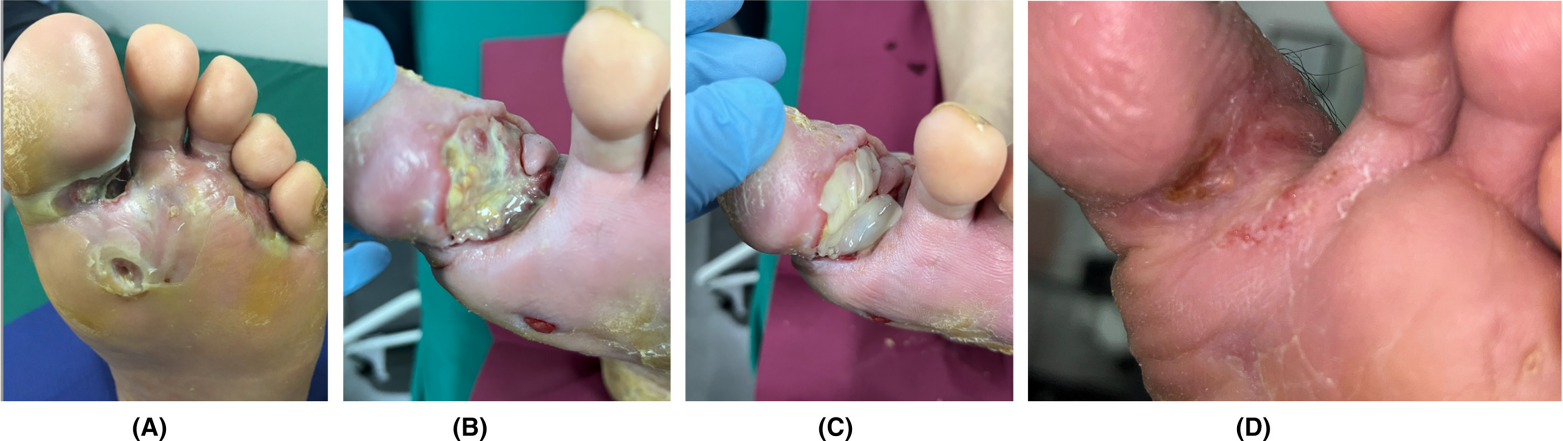
(A) Infected wound from a diabetic patient who stepped in a contaminated nail. (B) Conventional treatment and surgical debridement failed to improve the DFU, and an amputation was proposed as final treatment. (C) The patient refused and a NGR‐T was offered as last option. (D) After 8 weekly applications of L‐PRF membranes complete wound resolution could be obtained. Courtesy Dr. Yelka Zamora. Advanced Wound Care Center, San José, Costa Rica.

Four articles reported specifically on the healing of DFU, 1 RCT,^55^ 2 CCS,^56^, ^57^ and 1 prospective case series.^58^ Kartika et al.^55^ compared, over a 7 days period, a single application of 0.9% NaCl with the application of A‐PRF membranes or A‐PRF membranes combined with hyaluronic acid (HA). They observed an increased granulation index, higher levels of VEGF, and lower levels of IL‐6 in both A‐PRF groups, compared with NaCl, with A‐PRF + HA being superior to A‐PRF. In the article by Wang et al.,^56^ a comparison was made between the application of a mupirocin ointment plus rhEGF gel (twice/week) versus L‐PRF (weekly). L‐PRF scored significantly superior in wound size reduction as well as in the percentage of wounds with CR. The same group compared the effect of a Vaseline gauze with the use of L‐PRF (both applied every 2 days) in reaching the “standard wound condition” prior to skin grafting.^57^ L‐PRF reached these wound conditions significantly faster (shorter time to skin grafting, 6 vs. 12 days), with additional benefits including: less post‐operative infection, less graft necrosis, less ulcer recurrence, and lower need for foot amputation. Figure 2 shows the healing sequence of an infected DFU due to an accident with a contaminated nail.

**FIGURE 3:**
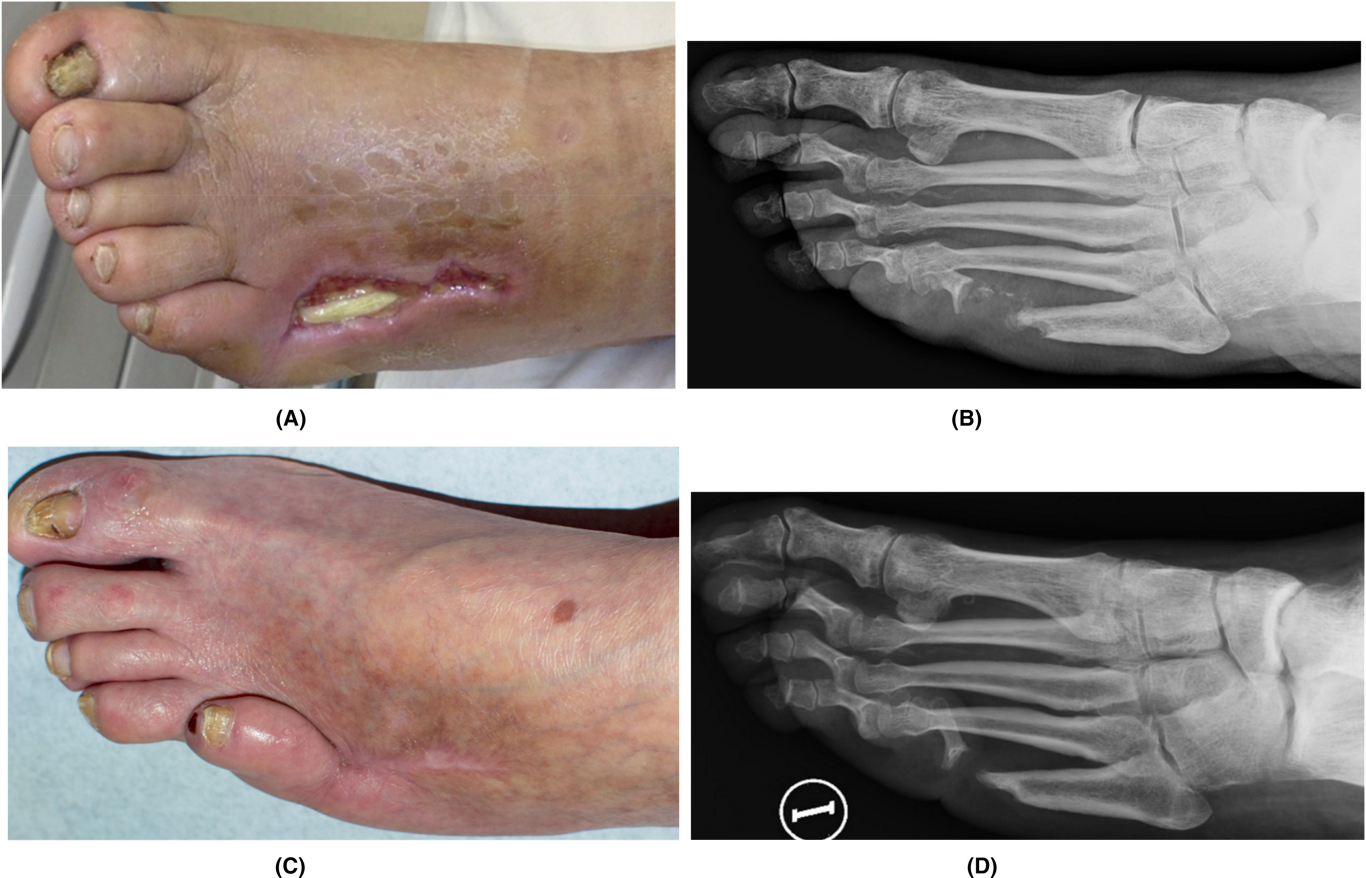
(A) A DFU with tendon exposure, osteomyelitis and fistula through the entire foot. (B) Radiograph at the start of the NGR‐T showing clear signs of osteomyelitis. The wound was completely closed after 7 weeks. (C) Control picture 5 years after wound closure without any recurrence of the ulcer, and with (D) the radiograph indicating a resolution of the osteomyelitis.

One prospective case series followed the healing of ulcerative osteomyelitis (gangrene) in 7 diabetic feet.^58^ A combination of: (i) a surgical debridement, (ii) the removal of dead bone particles, (iii) disinfection with H_2_O_2_ and iodopovidone, (iv) rinsing with A‐PRF exudate, and (v) application of A‐PRF membranes (face part) in the narrow but deep wound, eventually repeated a 2nd time, resulted in CR after 31.4 days in 6 of the 7 patients (1 patient died from coronary artery disease during the therapy), with no wound recurrence over the next 1 to 6 years. Figure 3 shows the healing of a DFU with osteomyelitis and a through‐and‐through fistula.

##### Venous leg ulcer

**FIGURE 4:**
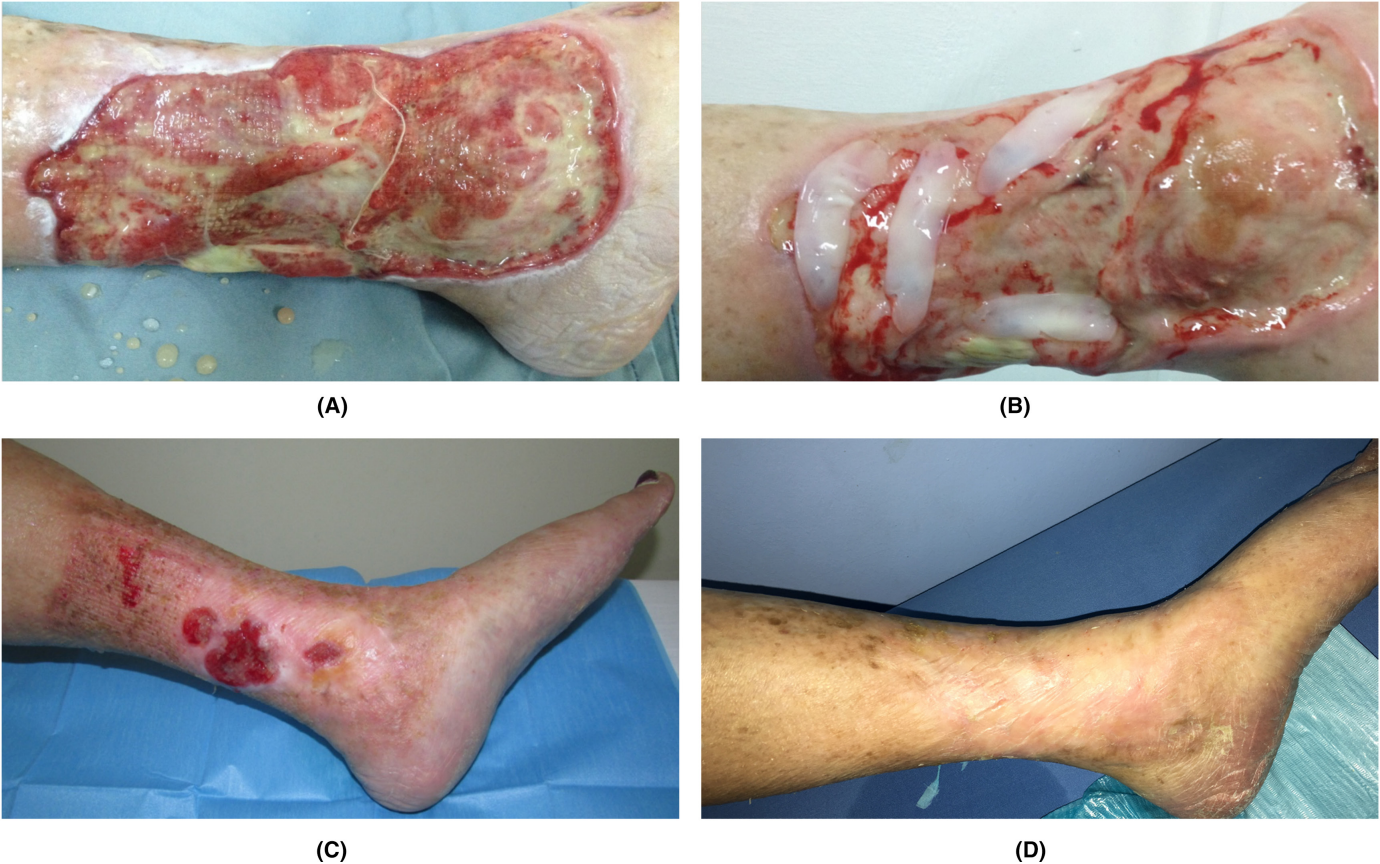
(A) A large, non‐healing, venous leg ulcer in a 72‐year‐old female, 12 years recalcitrant to regular advanced therapy. (B) Clinical image 1 week after first application of L‐PRF membranes with the formation of granulation tissue. (C) The wound significantly reduced in size within 8 weeks. (D) Wound close was reached after 3 months, and leg remained healthy as seen at the 1‐year follow‐up, without any recurrence. Courtesy Dr. Yelka Zamora. Advanced Wound Care Center, San José, Costa Rica.

Four RCTs compared the benefits of applying L‐PRF on VLU to other wound dressings.^59^, ^60^, ^61^, ^62^ Somani and Rai^59^ applied a saline dressing or L‐PRF clots weekly during 4 weeks. L‐PRF resulted in significantly more wound size reduction (86 vs. 43%) as well as CR (5/9 vs. 0/6). Goda^60^ compared the healing obtained with a paraffin gauze (every 2 days) to that of L‐PRF membranes and a paraffin gauze (weekly). The latter gave, after up to 8 weeks, more wound size reduction (100% vs. 62% for VLU ≤10 cm^2^ at week 4; 100% vs. 87% for VLU > 10 cm^2^ at week 7), and thus faster CR. Amato et al.^61^ compared the application of a CGF gel (heparin was added to the blood) plus a HA dressing with a HA dressing alone, and observed a significantly faster CR and less pain in the CGF group. Yuvasri and Rai^62^ explored the difference in wound healing between a weekly application of an Unna's paste or L‐PRF clots. After 4 weeks, the wound size reduction was rather similar, with however, more CR for the L‐PRF group (4/10 vs. 0/10). Figure 4 shows the impact of the local application of L‐PRF membranes on a recalcitrant VLUs with a non‐healing period of 12 years.

##### Leprosy

**FIGURE 5:**
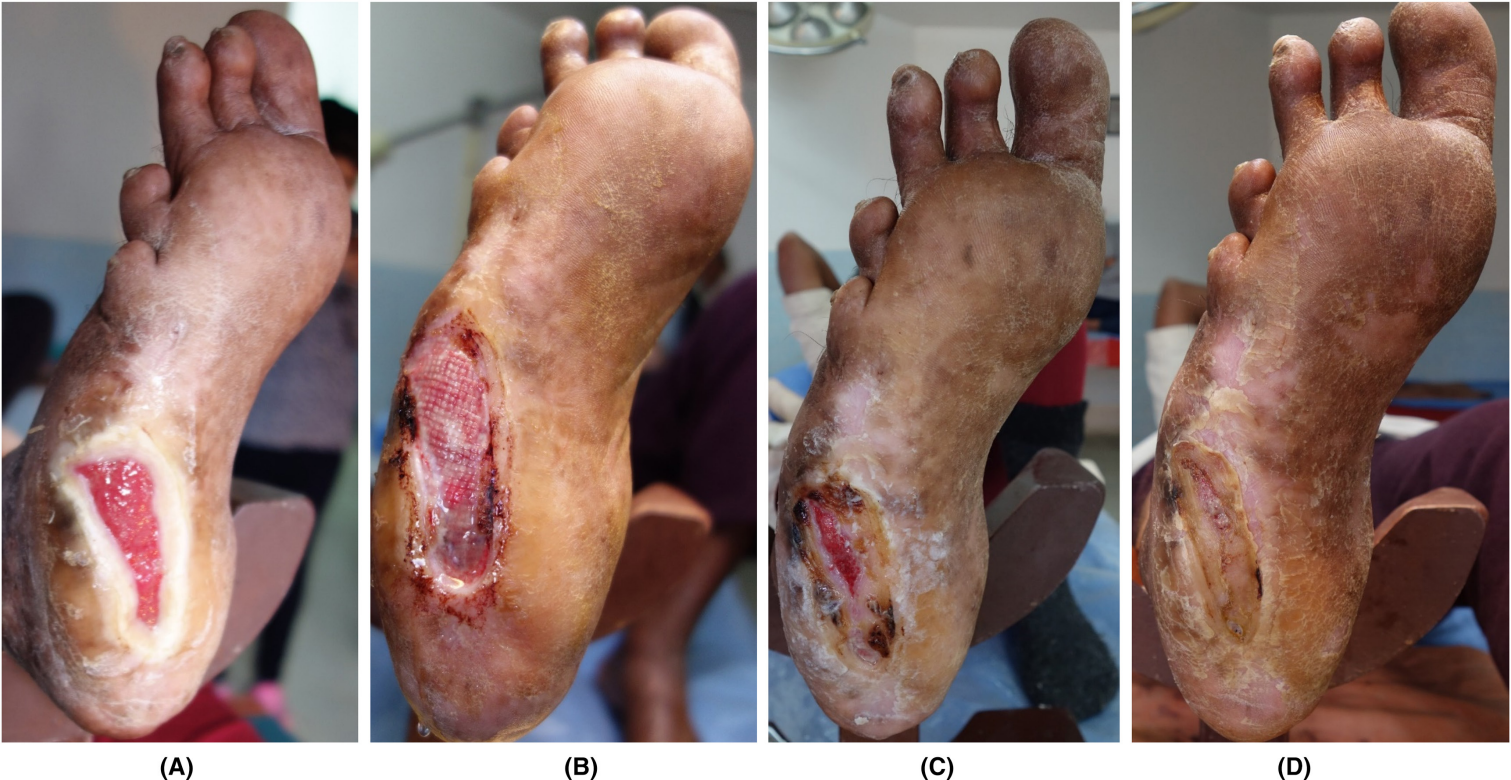
(A) A 69‐year‐old male patient with a leprosy plantar ulcer since 7 years, after a careful debridement to remove all dead tissue. (B–D) Progressive healing after NGR‐T with L‐PRF membranes after, respectively, week 3, week 5, and week 8. Case courtesy: Dr. Sushil Koirala (Punyaarjan Foundation) and Dr. Indra Napit (Anandaban Hospital TLM) Nepal.

Four articles specifically reported on non‐healing LU: one RCT^63^ and 3 prospective case series.^42^, ^64^, ^65^ Nagaraju et al.^42^ and Dorjay et al.^65^ applied L‐PRF membranes weekly on 2 to 12 months old LU. The first team reported a wound size reduction of 93.5% after 2 applications, and the mean number of L‐PRF applications to reach CR was 3. The second group obtained CR after 4 to 6 applications, and reported no recurrence up to 1 year after therapy. Figure 5 illustrates the healing capacity of L‐PRF in a large leprosy foot ulcers with a non‐healing history of 7 years.

Ghatge et al.^64^ applied L‐PRF clots weekly on trophic ulcers secondary to leprosy, and reached CR in 17/25 lesions after 6 weeks. Ratan et al.^63^ compared the weekly use of PRP (injection in the wound) with that of L‐PRF clots for primarily leprosy ulcers and could not find a significant difference between both approaches, neither in the rate of CR nor in the number of applications before CR.

#### 2.1.3 Additional info from case reports (≤3 patients)

Seven case reports^66^, ^67^, ^68^, ^69^, ^70^, ^71^, ^72^ introduce the “successful” use of L‐PRF for other chronic wounds such as: a non‐healing skin wound after excision squamous carcinoma, a facial pyoderma gangrenosum, DFUs with osteomyelitis, none healing erythema gangrenosum lesions, non‐healing VLU as Klinefelter syndrome, and a pressure injury post spinal cord surgery. The observations in these articles have of course to be confirmed in larger‐scale studies, preferably with a RCT design.

### 2.2 L‐PRF in other demanding extra‐oral wounds

In an RCT conducted by Chignon‐Sicard et al.,^73^ the healing of fresh palm wounds after fasciectomy, in patients with Dupuytren disease, was examined. In the test group (*n* = 33), L‐PRF membranes were applied and covered with non‐adherent dressings, in the control group (*n* = 31), Vaselitulle was used. The dressings were replaced every 2 days, paying attention not to disturb the ongoing wound healing. The authors observed that the L‐PRF group experienced less pain and required fewer dressing changes than the control group. Notably, despite having larger wounds, the L‐PRF group healed 5.4 days earlier than the control group (24 vs. 29 days).

Soyer et al.^74^ presented a single case of recurrent urethra‐cutaneous fistula following hypospadias repair. L‐PRF was placed between the fistula repair layer and the overlying skin suture. The repair was successful, without complications or recurrences at the 3‐month follow‐up. Inspired by Soyer's approach, Guinot et al.^75^ utilized L‐PRF membranes between the hypospadias repair (urethroplasty) layer and the skin coverage to reduce fistula formation (33 patients). In the control group (72 patients), a dorsal subcutaneous flap from the foreskin was used, as an alternative to L‐PRF, to cover the urethroplasty layer. Although there was no statistically significant difference in complication rates, L‐PRF membranes were easier to obtain, and the treatment was simpler to execute.

Several studies explored the impact of L‐PRF on the healing of tympanic membrane perforations^76^, ^77^, ^78^, ^79^, ^80^ and reported more perforation reduction (when compared with unassisted healing), as well as a better graft uptake with better improvement in air‐bone gap in case of surgery. A recent systematic review with meta‐analysis confirmed the beneficial impact of L‐PRF matrices.^81^

Zumstein et al. explored in 2 RCTs the adjunctive effect of L‐PRF during arthroscopic rotator cuff repair (transosseous‐equivalent tension band double‐row fixation) but were not able to identify major differences between sites with (*n* = 27) and without (*n* = 28) the use of L‐PRF.^82^, ^83^ This is, so far, the only indication where L‐PRF did not show any major advantage compared with a standard therapy.

Alviti et al.^84^ applied L‐PRF over the surgical site after Krackow's end‐to‐end suturing of ruptured Achilles tendons (*n* = 11) and observed superior functional improvements in terms of efficiency of motion when compared with sites without L‐PRF (*n* = 9). Zhang et al.^85^ compared the outcome of a tissue graft after tendon exposure, preparing the wound prior to grafting either with Integra or L‐PRF dressing in combination with negative pressure. The L‐PRF treatment resulted in a significant higher graft take rate and less pain.

Charrier et al.^86^ analyzed in a prospective case series (10 patients) the impact of L‐PRF clots for the soft‐tissue reconstruction after superficial or subtotal parotidectomy (intra‐operatively deposited upon the facial nerve trunk, and to fill the cavity). They reported a 100% satisfactory cosmetic outcome and the absence of adverse events.

Beneficial effects of L‐PRF during wound healing have been reported in several case series of endoscopic endonasal skull base surgery defect reconstruction,^87^, ^88^ bone healing of the skull base,^89^ reconstruction of the sphenoidal mucosal plane following trans‐sphenoidal endoscopic approaches,^88^ and dura mater.^90^

Finally, Vaheb et al.^91^ compared via an RCT the healing of split‐thickness skin graft donor sites (used to cover burns) using Vaseline petrolatum gauze with (*n* = 33) or without (*n* = 33) L‐PRF membranes to cover the wound underneath the dressing. The application of L‐PRF gave a faster healing and less pain, quite similar to the intra‐oral use of L‐PRF membranes as wound bandage after retrieving a free gingival graft or a subcutaneous connective tissue graft^92^, ^93^, ^94^, ^95^, ^96^, ^97^, ^98^, ^99^ as well as for the protection of large wounds.^100^, ^101^, ^102^, ^103^, ^104^

## 3 DISCUSSION

Our review on the use of L‐PRF in non‐healing chronic wounds (venous leg ulcers, diabetic foot ulcers, leprosy lesions and pressure ulcers) confirmed a beneficial impact. Data were collected on 533 chronic wounds, including 124 DFU, 196 VLU, 87 leprosy lesions, 10 pressure ulcers and 116 other types of chronic wounds (Table 1). Only one adverse event has been reported, in a patient with comorbidity and skin allergy, who, after an initial improvement, worsened resulting in an interruption of the L‐PRF application.^45^ In most patients, the ulcer was already presented for weeks/months/years, but failed on standard/conventional therapy. Nevertheless, in nearly all these chronic wounds, complete resolution could be obtained after L‐PRF applications, mostly on a weekly bases and over a period of 2 to 10 applications. In the 5 RCTs and 1 CCT, the application of L‐PRF was consistent statistically superior to the control therapy (standard/conventional dressing), with a faster healing, an earlier CR, and superior PROMs. Our observations are supported by recent systematic reviews.^105^, ^106^, ^107^, ^108^, ^109^, ^110^

The results of this review article should be interpreted with caution due to some limitations: (i) the heterogeneity between studies (e.g., study design, patient selection, pre‐operative improvement of medical condition of the patient, area and duration of ulcers, details on standard/conventional wound care prior to L‐PRF therapy, dosage and frequency of dressings, follow‐up period, etc.), (ii) a low number of RCT, especially those comparing the application of L‐PRF with other novel therapeutic approaches or on a combination with other procedures (e.g., negative pressure wound therapy (for pressure ulcer), hyaluronic acid (HA), etc.), (iii) difficulties in judging potential bias (e.g., randomization, blinding both on treatment allocation as well as in outcome assessment, etc.), (iv) low number of studies reporting on PROM (e.g., pain, analgesics, wound recurrence on a longer time), and (v) data on the economic burden for both the patient and the society. Further studies on the total expenses and comparison with standard wound care are warranted to indicate the final value of L‐PRF in the treatment of chronic wounds.

Chronic wounds indeed are associated with a major economic burden to society, comprising direct (medical and health care costs) and indirect costs (productivity losses, e.g., sick leave and early retirement).^28^, ^111^, ^112^, ^113^, ^114^, ^115^, ^116^ Treatment costs for chronic wounds are substantial and are estimated to account for approximately 2%–3% of the total healthcare expenditure in developed countries.^117^, ^118^, ^119^, ^120^, ^121^, ^122^ The need to prevent chronicity by improving healing rates and times through innovation and high‐quality research is clearly evident.

The question of whether a treatment with L‐PRF will substantially reduce the financial impact of chronic wound care on patients, as well as the healthcare system, is difficult to answer objectively. Many factors must be included in the equation:

- The direct costs:
  - The need for more staff,
  - The costs of an L‐PRF treatment (time for blood draw (3–5 min), centrifugation (12 min), compression of L‐PRF clots to membranes (optional, 5 min), and application of L‐PRF and wound dressing (4 min)),
  - The repetitive collection of whole blood,
  - The investment in the equipment (including the centrifuge),
  - The consumables (very low cost), and
  - The duration of the treatment.
- The indirect gains
  - Lower costs for home self‐dressing, medical examinations, and possible hospitalizations and
  - Shorter treatment time because of faster wound healing (closure) and less wound recurrence.
- Additional aspects to consider:
  - The variation between patients; most patients belong to the category of geriatric patients and may have numerous co‐morbidities that influence the course of the wound and interfere with the healing pathway.

Even if the dressing provided with L‐PRF has a higher unit cost than the standard dressing, the total cost of the treatment is very advantageous since the number of dressings necessary for healing is generally largely reduced. As such, the final care costs and workload of health professionals will decrease, including a shortening of the patient's hospitalization period. To confirm this hypothesis, “objective” studies, including the economic aspects, are urgently required.

A recent systematic review with a definition for wound chronicity (duration ≥ 3 weeks) aimed to examine the humanistic burden for patients suffering from chronic wounds by applying Health‐related quality of life (HRQoL) measures.^121^ Patients with chronic wounds most frequently reported lower HRQoL scores in the domains related to physical pathologies, i.e., physical role, physical functioning, equivalent to mobility, but also for emotional‐ and mental health domains, as well as for the domains of bodily pain and discomfort. Long wound duration and/or large wound size, as well as pain were correlated to poorer HRQoL scores. The inferior HRQoL observed for patients with chronic wounds is worst for physical pathologies and is similar or markedly lower in some HRQoL domains when compared with other chronic conditions, e.g., chronic obstructive pulmonary disease and cardiovascular disease.^121^

**FIGURE 6:**
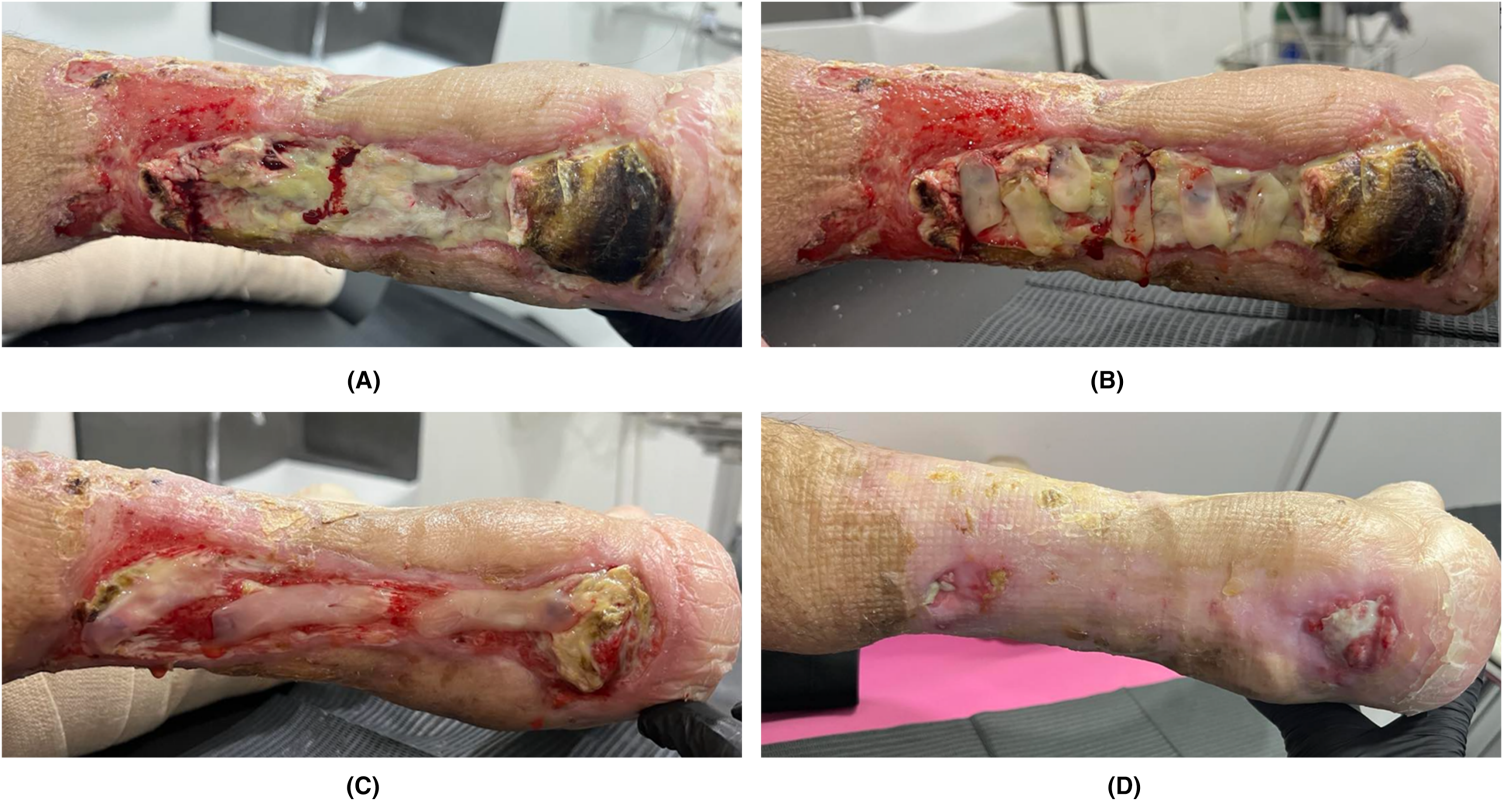
(A) A 58‐year‐old female diabetic patient was suffering from a large *complex wound* which did not correspond to any advanced wound care. (B) L‐PRF membranes were applied weekly, following the NGR‐T concept. (C) The wound gradually reduced in size, as seen on the pictures after 13 weeks, and after 17 weeks (D) a nearly complete resolution could be obtained. Courtesy Dr. Yelka Zamora. Advanced Wound Care Center, San José, Costa Rica.

**FIGURE 7:**
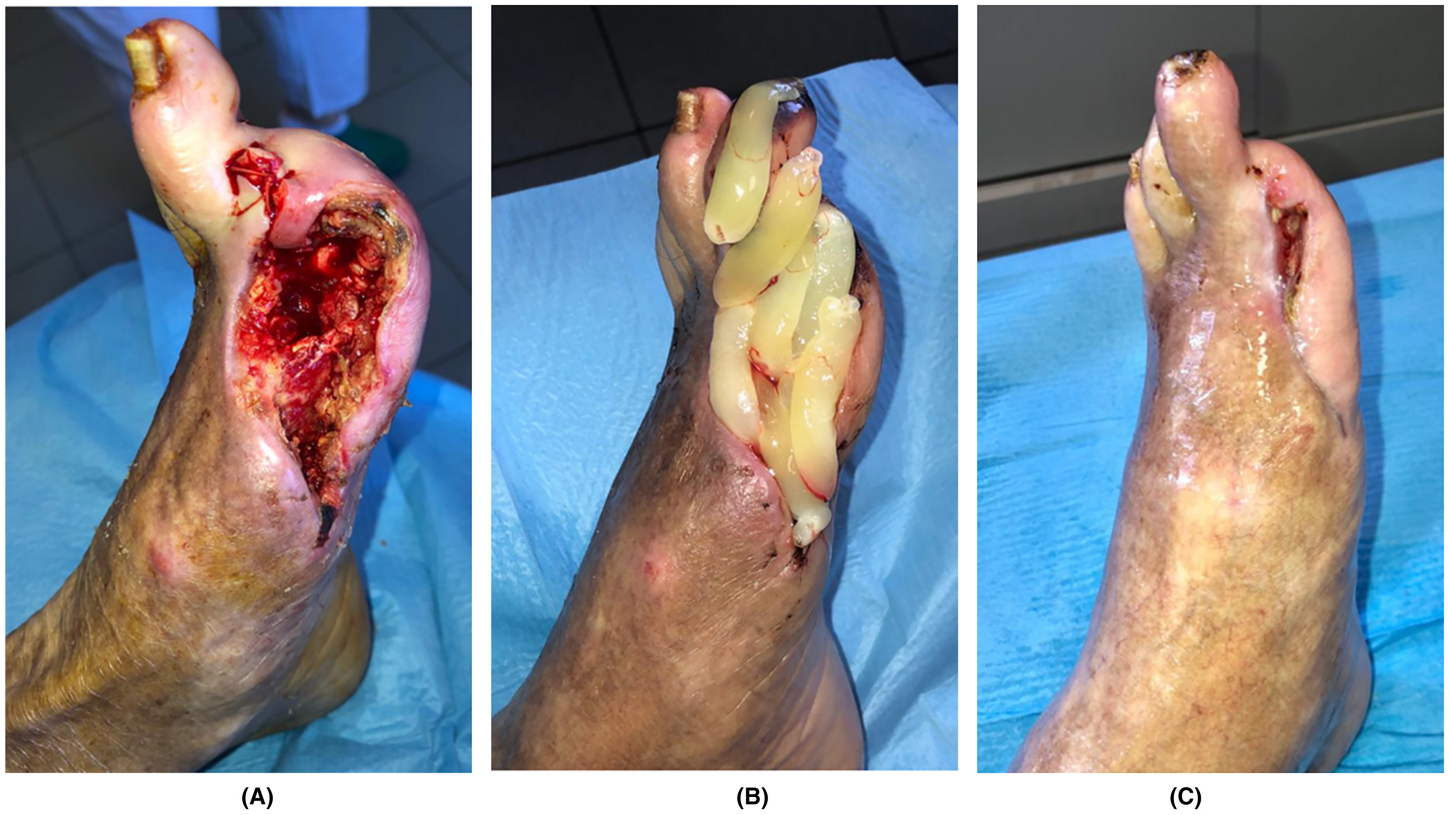
A 82‐year‐old man developed a chronic ischemia after the amputation of the big toe and neighboring toe. (A) After a careful debridement to remove all death tissue, (B) NGR‐T was initiated via the application of 8 L‐PRF clots. (C) Complete wound resolution could be obtained within 10 weeks. Courtesy Dr. Enrico Rescigno, vascular surgeon, Genoa, Italy.

**FIGURE 8:**
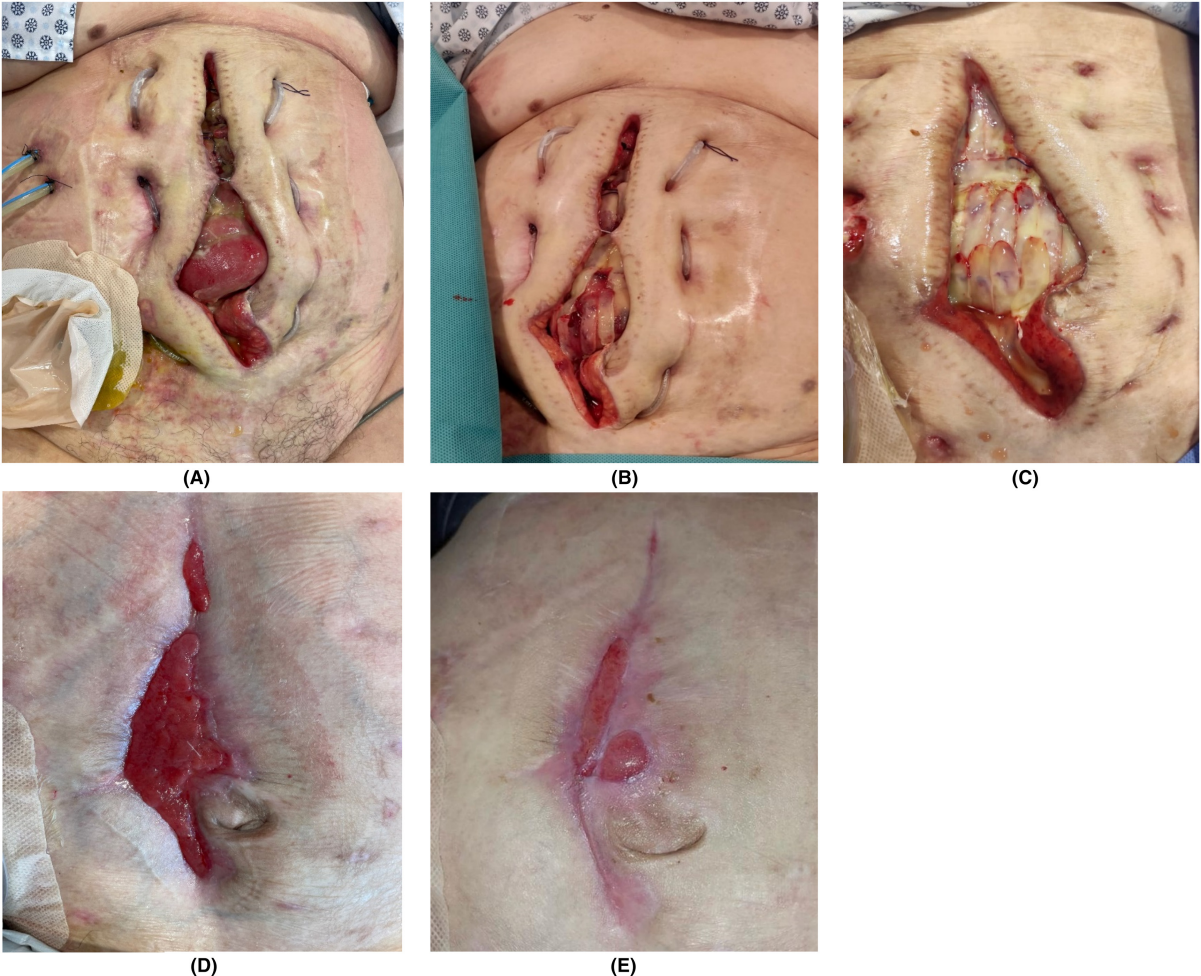
NGR‐T of large wound dehiscence after colectomy. (A) Wound dehiscence and evisceration, (B) Clinical condition after two L‐PRF applications within 2 weeks, (C) Condition after 6 weeks with reapplication of L‐PRF membranes, (D) Wound completely filled with granulation tissue after 9 weeks and last application of L‐PRF membranes. (E) Nearly complete wound closure at last follow‐up visit. Case courtesy: Dr. Franco Innocenti, Associate Professor of Surgery, Universidad de Concepción & Dr. Diego A. Pinto, resident in orthopedic surgery, Hospital Traumatológico, Universidad de Concepción, Chile.

**FIGURE 9:**
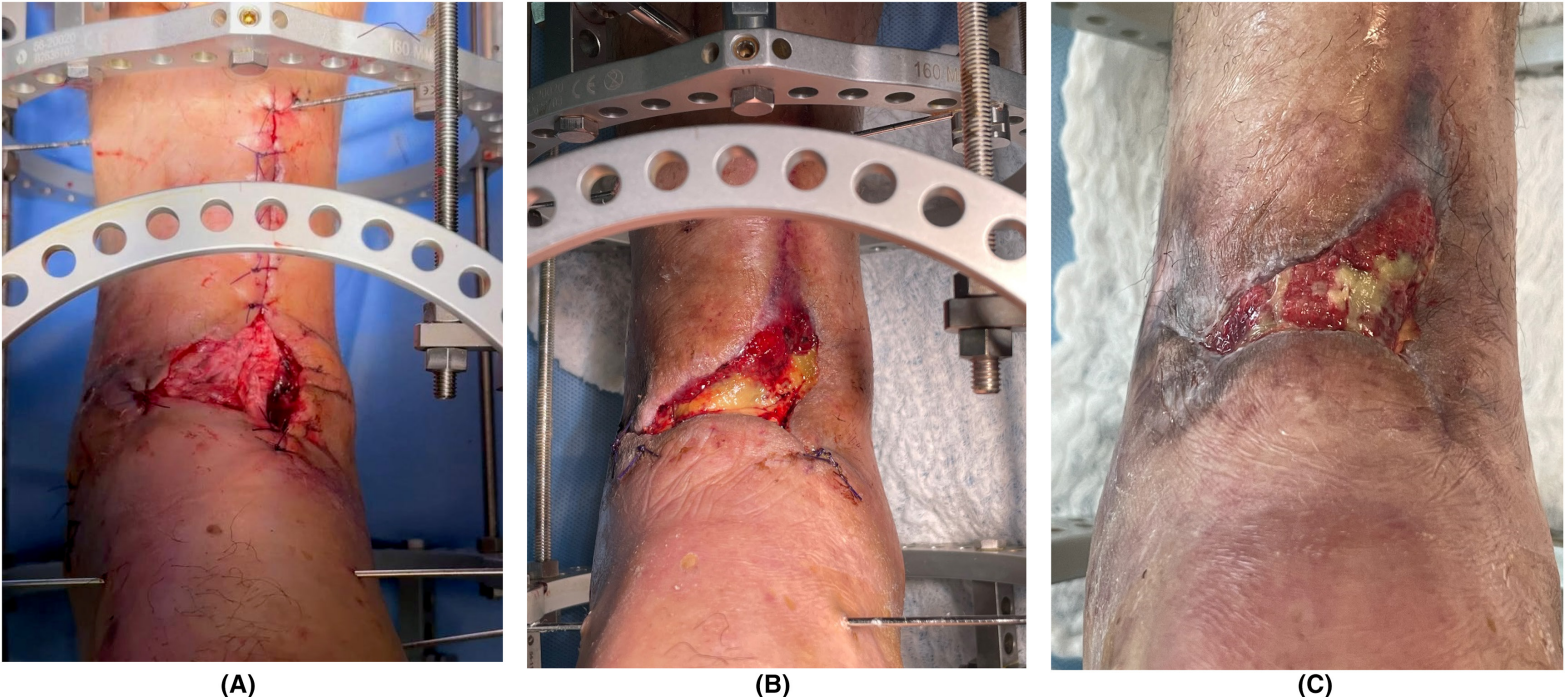
NGR‐T of soft tissue necrosis (including bone and tendon exposure) after treatment of a tibial pilon fracture (A). (B) Clinical picture after 3 weekly L‐PRF applications. (C) Clinical view after 8 weeks with complete bone and tendon coverage; the wound is now ready for dermo‐epidermal grafting which progresses without complications. Case courtesy: Dr. Cesar Cisterna, Orthopedic Surgeon, Hospital Traumatológico, Concepción and Dr. Diego A. Pinto, resident in orthopedic surgery, Hospital Traumatológico, Universidad de Concepción, Chile.

Following cases illustrate the strength of L‐PRF membranes. Figures 6 and 7 show the healing of 2 large chronic wounds with a complex multi‐factorial etiology, with nearly complete resolution after 17 and 10 weeks, respectively. Figure 8 shows a surgical complication after a colectomy in a 74‐year‐old lady with a history of COPD and colon cancer, with the separation of the wound edges at the entire suture line together with the protrusion of the internal organs (evisceration). Microbiological examination of the wound was positive for *Candida* spp. and *Klebsiella BLEE*. After five applications of L‐PRF membranes in a sterile manner as wound dressing over a 9 weeks period, wound closure was nearly obtained, without any complications. Figure 9 corresponds to a 60‐year‐old healthy male patient with a tibial pylon fracture as a consequence of an accident. After fracture treatment soft tissue necrosis occurred with loss of anterior coverage, and bone and tendon exposure. Wound closure was attempted with V.A.C. (Vacuum Assisted Closure) without positive response. A weekly application of L‐PRF membranes was initiated, during the first 4 weeks under spinal anesthesia, afterwards on an outpatient basis until complete bone and tendon coverage was achieved (after 8 weeks), followed by dermo‐epidermal grafting.

Nowadays, this treatment is called “Natural Guided Regeneration‐Therapy” (NGR‐T) because it only uses the patient's own biological components without adding anything else. The therapy by itself is not difficult, but the authors' advice is to follow the included clinical recommendations, based on the data from Table 1 and on the clinical experience of several authors of this article (NP, SK, ER, and YZ), each with more than 10 years of experience in the treatment of extra‐oral wounds with L‐PRF.

## 4 GENERAL CONCLUSION

The literature suggests that the repeated application of L‐PRF (clots or membranes) has a significant adjunctive impact on the healing of various acute and chronic extra‐oral wounds, including the proportion of wounds achieving complete resolution (100% re‐epithelialization), reduction in wound size (area and/or volume), time to complete resolution and healing rate, as well as on patient's quality of life by increasing their ability to engage in daily activities, and by reducing their immobility and dependence. Naturally, one should be aware that any treatment with L‐PRF should also include disease management to guarantee a successful clinical outcome, speed up the healing process, and prevent ulcer recurrence.

Moreover, clinicians and researchers should understand the true nature of L‐PRF and the specifics of L‐PRF therapy (i.e., protocol of preparation, e.g., choice of centrifuges and tubes), as well as of the clinical application. Researchers should design bias‐minimized randomized clinical trials and recognize superfluous brand competitions that delay sound progress. Long‐term follow‐up studies and randomized controlled trials will help further clarify the effectiveness, safety, and best use of L‐PRF in treating various chronic wounds. This will enable applying Naturally Guided Regeneration Therapy (NGR‐T) more widely and safely in clinical settings.

As the world's population ages and the prevalence of chronic wounds increases, L‐PRF can play a crucial role in alleviating the burden these debilitating diseases place on healthcare systems worldwide. By investing in further studies and research on L‐PRF, the medical community can advance the understanding of this promising Natural Guided Regeneration Therapy and work to provide better care for patients suffering from chronic and acute wounds.

## 5 RECOMMENDATIONS

### 5.1 Step‐by‐step protocol for L‐PRF use in DFU, VLU, PU, and CU

The treatment strategy, highlighted below, for chronic wounds including diabetic foot ulcers (DFU), venous leg ulcers (VLU), pressure ulcers (PU), and complex ulcers (CU) includes a number of similar steps, but also some wound‐type‐specific steps. The wound‐type‐specific steps are indicated with their abbreviation. It is extremely important to start with an evaluation of a patient's medical condition and to improve his/her general status (verifying underlying systemic conditions, medication, nutrition, etc.) before the therapy.

#### 1st appointment

**Table:** 

1. Gently clean the wound through irrigation with a standard saline solution to remove all exudate. The cleaning can be finalized by irrigation with a non‐cytotoxic antimicrobial solution (preferably also containing a surfactant). One can also use gauze embedded in this solution to clean the wound gently	
2. Disinfect the wound, and area around the wound to prevent contamination of the gloves/surgical material/L‐PRF membranes when touching this area during the following treatment	
3. Perform a *superficial debridement* to remove necrotic material, eschar, devitalized tissue, or any other type of bioburden from the wound (provide some bleeding points), including wounds with tunnels and/or cavities, to promote wound healing. Profound debridement *should be avoided* because of its negative impact!	
4. Slightly undermine the wound borders to create an envelope (±4 mm in width) into which the L‐PRF membranes can be slid to force and stimulate epithelium to adhere and migrate over the membranes instead of underneath. The migration of the epithelium does not occur immediately if the wound is profound	
5. Inject small amounts of L‐PRF *exudate* (*not* to be confused with liquid fibrinogen) in the wound area and the wound periphery. Use a thin needle (>30 gauge). The exudate is rich in growth factors and has antibacterial properties, and its injection will increase bleeding spots to supply the covering membranes with blood	
6. Apply L‐PRF *membranes* (not the clots) over the entire wound area, starting from the periphery towards the center of the wound (slid them into the envelope under the wound borders)	
7. Only in deeper areas of the wound should several layers of membranes be applied up to the level of the wound borders to speed up the refill of these areas with granulation/connective tissue	
8. Cover the wound with a non‐adhering knitted primary dressing (e.g. cellulose acetate impregnated with a petrolatum emulsion). It should (i) be conformable to the wound bed, (ii) have the ability to stay in situ over wear time, (iii) transmit wound exudate to the secondary dressing, and (iv) give minimal trauma on removal	
9. Cover this dressing and seal the periphery of the wound with a plastic film to ensure, in combination with the previous dressing, a moist environment to protect the L‐PRF membranes from dehydration. Dehydration should indeed be avoided at all times! This film should provide a waterproof, sterile barrier to external contaminants including liquids, bacteria, and viruses	
10. Apply a dry dressing to capture the typical exudate of the wound that usually increases during the first application. At the lower point of the wound, the plastic film will automatically peel off when wound exudate accumulates. The exudate will leak out at this point. The dry dressing can be changed when needed (e.g., to absorb additional exudate to avoid bad odors) without disturbing the wound dressing underneath	
DFU CU PU	Patients should avoid pressure and movement on the wound site specially the first 3 days after treatment In case of foot deformities (e.g., Charcot's foot), the patient should wear special shoes or completely avoid stepping on the affected foot for at least a week
VLU	Always apply an elastic bandages as standard compressive therapy
PU	Patients should avoid pressure and movement on the wound site specially the first 3 days after treatment

### 5.2 Step‐by‐step protocol for L‐PRF use in “leprosy planter ulcer” (LU)

The treatment strategy highlighted below is exclusive for leprosy‐induced chronic ulcer (LU).

#### 1st appointment

**Table:** 

1. Gently clean the wound through irrigation with a standard saline solution to remove all exudate. The cleaning can be finalized by irrigation with a non‐cytotoxic antimicrobial solution (preferably also containing a surfactant). One can also use gauze embedded in this solution to clean the wound gently	
2. Disinfect the area around the wound to prevent contamination of the gloves/surgical material/L‐PRF membranes when touching this area during the following treatment	
3. LU generally have hard callus formation around the wound periphery. Hence it is necessary to remove such hard callus with a surgical blade and make it get bleeding points if possible. Then one can perform a *superficial debridement* to remove necrotic material, eschar, devitalised tissue, or any other type of bioburden from the wound (provide some bleeding points), including wounds with tunnels and/or cavities, to promote wound healing. Profound debridement *should be avoided* because of its negative impact!	
4. Slightly undermine the wound borders to create an envelope (±4 mm in width) into which the L‐PRF membranes can be slid to force and stimulate epithelium to adhere and migrate over the membranes instead of underneath. The migration of the epithelium does not occur immediately if the wound is profound	
5. Apply L‐PRF *membranes* (not the clots) over the entire wound area, starting from the periphery towards the center of the wound (slid them into the envelope under the wound borders)	
6. Only in deeper areas of the wound, several layers of membranes should be applied up to the level of the wound borders to speed up the refill of these areas with granulation/connective tissue	
7. Cover the wound with a non‐adhering knitted primary dressing (e.g. cellulose acetate impregnated with a petrolatum emulsion). It should (i) be conformable to the wound bed, (ii) have the ability to stay in situ over wear time, (iii) transmit wound exudate to the secondary dressing, and (iv) give minimal trauma on removal	
8. Apply a dry dressing to capture the typical exudate of the wound that usually increases during the first application. The dry dressing can be changed when needed (e.g., to absorb additional exudate, to avoid bad odors) without disturbing the wound dressing underneath	
LU	Patients should avoid pressure and movement on the wound site, especially the first 3 days, and should wear special shoes or completely avoid stepping on the affected foot for at least a week. Patients should protect other areas of the foot from dryness and apply suitable moisturizers (e.g., paraffin wax dermal ointment).

### 5.3 2nd and following appointments (all wounds) preferably every week, at least every 2nd week)

**Table:** 

1. Gently remove all dressing and clean the wound through abundant irrigation with a standard saline solution, finalizing by irrigation with a non‐cytotoxic antimicrobial solution. Eventually, clean the wound gently with a gauze embedded with this product
2. Check the integrity of the L‐PRF membranes. Those that are attached to the wound surface or those that are becoming granulation‐type tissue should not be removed
3. Loose L‐PRF membranes, necrotic tissue, and eschar, however, should be removed
4. Repeat steps 4 to 10 (see previous page), *avoiding* any unnecessary or profound debridement
5. Repeat this procedure weekly until complete coverage of the wound with healthy epithelium. At the end of the treatment, longer time intervals (e.g. 2 weeks) can be taken into consideration The need for painkillers should reduce significantly after the first applications of the L‐PRF membranes The bad smell of the wound should be replaced by the typical smell of using L‐PRF membranes

### 5.4 Aftercare for all wounds

**Table:** 

✓ Inform the patient about measures to prevent the recurrence of the ulcer!	
✓ The patient should regularly apply a dermal ointment on the regenerated skin to keep this skin soft and lubricated.	
✓ The patient should protect the area from direct sun exposure and always use sunscreen.	
✓ The patient should protect the healed site from scratches and external shocks!	
DFU CU VLU	Inform the patient on the importance of correct medication
VLU	Instruct the patient to use daily a compressive therapy
PU	Instruct the patient to prevent pressure points to the healed site
LFU	Inform the patient on the importance of correct medication. Instruct the patient to prevent pressure points to the healed site. The patient should regularly apply paraffin wax or a dermal ointment on the other area of the foot to keep the skin soft and lubricated. Moreover, proper footwear is essential

## 6 GENERAL RECOMMENDATIONS FOR WOUND TREATMENT WITH L‐PRF

**Table:** 

✓ Before any intervention of the wound, its etiology should be identified, and a causal therapy should be started!
✓ It can be useful to measure and record the length, width, and depth of the wound. Pictures of the wound with a reference (e.g., measuring rod ruler, millimeter paper) can be useful to follow the evolution of the wound
✓ All chronic wounds have a biofilm and are contaminated with different bacteria. Any signs of infection should be taken into consideration!
✓ It is advisable to run a bacterial culture from the wound before the start of the L‐PRF therapy!
To reduce the chance of major wound infection it is suggested to: Prepare a sterile drape around the wound, Use surgical gloves and a surgical mask, and To disinfect not only the wound but also the area around the wound to prevent transmission of infection from this area to the wound

## 7 WOUND INFECTION

**Table:** 

✓ All chronic wounds are contaminated with different bacteria. Wound infections during the L‐PRF treatment are, however, rare!
✓ When the first signs of infection are seen, an even more thorough *disinfection* should be undertaken via, for example, the application of a gauze embedded with a non‐cytotoxic antimicrobial solution to the infected area for several minutes
✓ When applying the L‐PRF membranes, make sure that the face of the membranes (the red part of the membrane that initially was in contact with the red blood cells) is applied towards the suspected area. The leukocytes in the face area can, as such, more easily reach the infection.
✓ Antibiotic therapy is not recommended unless a major infection is observed. Specific systemic antibiotics should be given concomitantly with the L‐PRF therapy, based on the outcome of a bacterial culture!

## CONFLICT OF INTEREST STATEMENT

All authors declare no conflict of interest regarding this chapter, although some may have received research support from implant companies such as Dentsply Sirona, Straumann, and Henry Schein.

## Data Availability

Data sharing not applicable to this article as no datasets were generated or analysed during the current study.
